# Reference values of normal abdominal aortic areas in Chinese population measured by contrast-enhanced computed tomography

**DOI:** 10.3389/fcvm.2022.950588

**Published:** 2022-09-13

**Authors:** Xiang Wang, Shasha Jin, Qing Wang, Jiawei Liu, Fei Li, Haiwei Chu, Dexing Zheng, Xiaolong Zhang, Jianrong Ding, Jingli Pan, Wenjun Zhao

**Affiliations:** ^1^Department of Vascular Surgery, Taizhou Hospital of Zhejiang Province, Wenzhou Medical University, Linhai, China; ^2^Department of Information and Technology Center, Taizhou Hospital of Zhejiang Province, Wenzhou Medical University, Linhai, China; ^3^Department of Central Laboratory, Taizhou Hospital of Zhejiang Province, Wenzhou Medical University, Linhai, China; ^4^Department of Radiology, Taizhou Hospital of Zhejiang Province, Wenzhou Medical University, Linhai, China

**Keywords:** abdominal aortic area, reference value, contrast-enhanced computed tomography, abdominal aortic aneurysm, diameter

## Abstract

**Objective:**

To generate reference values of the normal areas of the abdominal aorta at various levels among Chinese people and to explore the factors that may promote the expansion of the abdominal aorta.

**Methods:**

The areas of normal abdominal aortas were gauged at various levels based on inner-to-inner measurements in 1,066 Chinese adult patients (>18 years) without the abdominal aortic disease. The areas of subphrenic abdominal, suprarenal abdominal, infrarenal abdominal, and distal abdominal aortas were measured. The demographic and clinical characteristics were collected into a specifically designed electronic database. Multivariable linear regression was used to analyze the potential risk factors promoting the expansion of the abdominal aorta.

**Results:**

In males, the median areas of the subphrenic abdominal aorta, suprarenal abdominal aorta, infrarenal abdominal aorta, and distal abdominal aorta were 412.1, 308.0, 242.2, and 202.2 mm^2^, respectively. In females, the median areas of the subphrenic abdominal aorta, suprarenal abdominal aorta, infrarenal abdominal aorta, and distal abdominal aorta were 327.7, 243.4, 185.4, and 159.6 mm^2^, respectively. The areas of the abdominal aorta at different levels were larger in males than in females and increased with age. Multiple linear stepwise regression analysis showed that the subphrenic abdominal aortic area was significantly related to age (β = 0.544, *p* < 0.001), sex (β = 0.359, *p* < 0.001), and hypertension (β = 0.107, *p* < 0.001). Suprarenal abdominal aortic area was related to age (β = 0.398, *p* < 0.001), sex (β = 0.383, *p* < 0.001), history of smoking (β = 0.074, *p* = 0.005), and hypertension (β = 0.111, *p* < 0.001). The infrarenal abdominal aortic area was correlated with age (β = 0.420, *p* < 0.001), sex (β = 0.407, *p* < 0.001), and history of smoking (β = 0.055, *p* = 0.036). The distal abdominal aortic area was correlated with age (β = 0.463, *p* < 0.001), sex (β = 0.253, *p* < 0.001), and hypertension (β = 0.073, *p* = 0.013).

**Conclusion:**

The abdominal aortic areas at different levels were larger in males than in females. Aging, hypertension, and smoking prompt the expansion of abdominal aorta.

## Introduction

Abdominal aortic aneurysm (AAA) is generally defined as an enlargement of the abdominal aorta with a maximum diameter ≥ 3.0 cm or as a focal dilation ≥ 1.5 times the diameter of the normal aorta. Surgical repair is recommended for patients with AAA with a maximum diameter > 5.5 cm in males and 5.0 cm in females (1). Larger diameter aneurysms have a higher risk of rupture (2). Hence, at present, the maximum diameter plays an important role in the management of an AAA.

However, some scholars have also raised questions about the use of maximum diameter in AAA management. They demonstrated that AAA-related complications, neck-related events, and secondary interventions were not associated with the largest AAA diameter in patients with AAA (3). Therefore, they considered that the maximum abdominal aortic diameter may not be the best indicator for AAA management. Moreover, these studies have suggested other indicators that are more sensitive in predicting AAA progression, such as c-reactive protein (CRP), insulin-like growth factor 1 (IGF-1), antiphospholipid (APL) antibodies, and matrix metalloproteinase-9 (MMP-9) (4). One of the most discussed surrogates was the flow lumen area of an AAA. The shapes of cross-sections of an AAA perpendicular to its center lumen line are not always circular. Hence, the maximum diameter cannot fully represent the morphology of the AAA. Current studies have shown that the flow lumen area is more robust in predicting the rupture risk of AAAs (5). Therefore, the normal area of the abdominal aorta needs to be defined to provide a detailed reference for diagnosing an AAA and help make proper clinical decisions.

Given that few studies have reported normal abdominal aortic areas at different levels, this study gauged the areas of the abdominal aorta in Chinese adults without AAA at various levels and explored the factors that may promote the expansion of the abdominal aorta.

## Materials and methods

### Study sample

A retrospective study was performed to determine the normal abdominal aortic areas at different levels in hospitalized adult patients (>18 years) without AAA who had undergone abdominal contrast-enhanced computed tomography (CT) scans from July 2021 to December 2021. The exclusion criteria were a history of aortic diseases (aneurysm, dissection, and intramural hematoma).

### Imaging technology and information collection

A 256-row CT or a 64-row CT (Revolution Apex CT or Discovery CT 750 HD CT, GE Healthcare) was used for examination. Scanning parameters were as follows: the tube voltage,100–120 kv; tube current, 200–250 mA; thickness,5 mm; gantry rotation time, 0.5 s; and the scanning range was from diaphragm top to pubic symphysis.

**FIGURE 1:**
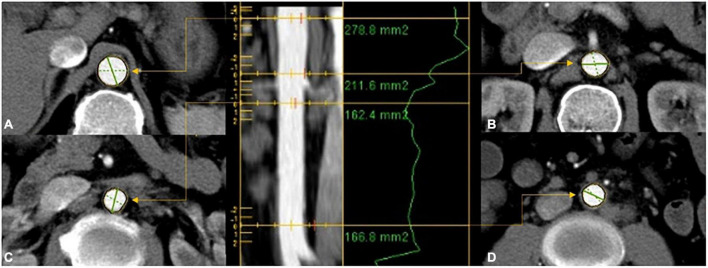
The areas of aortic cross-sections were gauged based on inner-to-inner measurements at four levels: **(A)** proximal abdominal aorta; **(B)** suprarenal abdominal aorta; **(C)** infrarenal abdominal aorta; **(D)** distal abdominal aorta.

In total, two experienced radiologists independently investigated the CT images captured from 1,066 patients independently using the Vessel IQ software on an offline workstation (Advantage Workstation 4.7, GE Healthcare). The areas of aortic cross-sections perpendicular to the aorta’s center lumen line were gauged based on inner-to-inner measurements at four levels: the subphrenic abdominal aorta (Figure 1A), suprarenal abdominal aorta (Figure 1B), infrarenal abdominal aorta (Figure 1C), and distal abdominal aorta (Figure 1D). For each level of every aorta, the average area was calculated and subjected to subsequent analysis.

The demographic and clinical characteristics were collected into a specifically designed electronic database.

The body mass index (BMI) and body surface area (BSA) was calculated by the following formula.


$$\text{BMI}=\frac{\mathrm{Weight}\,\left(\mathrm{kg}\right)}{{\mathrm{Height}}^{2}\,\left(m^{2}\right)}$$

BSA=0.007184×W⁢(kg)⁢0.425×H⁢(cm)⁢0.725

### Statistical analysis

Statistical analysis was performed using statistical package for social sciences (SPSS 26.0). Continuous variables data are reported as the mean ± standard deviation. The patients were grouped by age with an interval of 10 years. For each group, different percentiles (5th, 25th, 50th, 75th, and 95th) of abdominal aortic areas were calculated. Gender-based subgroup analysis was performed. Categorical and ordinal data are reported as frequencies and percentages. Linear regression was used to determine potential risk factors promoting the expansion of the abdominal aorta. Comparisons between groups were performed by Student’s *t-*tests for normally distributed data with homogeneous variances or by non-parametric Mann–Whitney Wilcoxon test. Kruskal-Wallis test followed by Dunn’s multiple comparisons test were used to compare data among groups of ≥ 3 with non-normal distributions. A two-sided, *p-*value < 0.05 was considered statistically significant.

## Results

### Patient characteristics

From July 2021 to December 2021, 1,087 adult patients have undergone abdominal contrast-enhanced CT scans. According to the exclusion criteria, 21 patients were excluded (15 patients had AAA, 4 patients had abdominal aortic intramural hematoma, and 2 patients had abdominal aortic dissection). At last, 1,066 patients were included in our study.

The average age of all 1,066 patients was 61.32 ± 14.61 years (range from 18 to 95 years). 608 patients (57.0%) were male, with an average age of 62.08 ± 14.50 years (range from 18 to 95 years), and 458 patients (43.0%) were female, with an average age of 60.31 ± 14.71 years (range from 18 to 93 years). The characteristics of the patients are presented in Table 1.

**TABLE 1:** Clinical characteristic of 1,066 subjects.

Variables	Male	Female	Overall
*N*	608 (57.0)	458 (43.0)	1,066
Age	62.08 ± 14.50	60.31 ± 14.71	61.32 ± 14.61
Weight (kg)	66.10 ± 11.07	57.04 ± 9.45	62.28 ± 11.37
Height (cm)	167.56 ± 6.47	156.79 ± 6.33	162.92 ± 8.36
BMI	23.51 ± 3.73	23.21 ± 3.59	23.43 ± 3.58
BSA	24.56 ± 4.71	19.83 ± 3.64	22.58 ± 4.86
History of smoking (%)	182 (29.9)	2 (0.4)	184 (17.3)
History of drinking (%)	103 (16.9)	1 (0.2)	104 (9.8)
Hypertension (%)	182 (29.9)	133 (29.0)	315 (29.5)
Diabetes (%)	65 (10.7)	61 (13.3)	126 (11.8)
Cardiovascular disease (%)^a^	55 (9.0)	37 (8.1)	92 (8.6)
Digestive system disease (%)^b^	512 (84.2)	355 (77.5)	867 (81.3)
Respiratory system disease (%)^c^	139 (22.9)	75 (16.4)	214 (20.1)
Urinary system disease (%)^d^	90 (14.8)	42 (9.2)	132 (12.4)
Cerebrovascular disease (%)^e^	54 (8.9)	40 (8.7)	94 (8.8)

Continuous variables data are given as mean ± standard deviation.

BMI, body mass index; BSA, body surface area.

^a^Mainly ischemic heart disease.

^b^Mainly digestive cancer.

^c^Mainly chronic obstructive pulmonary disease.

^d^Including renal impairment and urinary cancer.

^e^Including transient ischemic attack, reversible ischemic neurologic deficit, and stroke.

### Reference values for abdominal aortic areas

**FIGURE 2:**
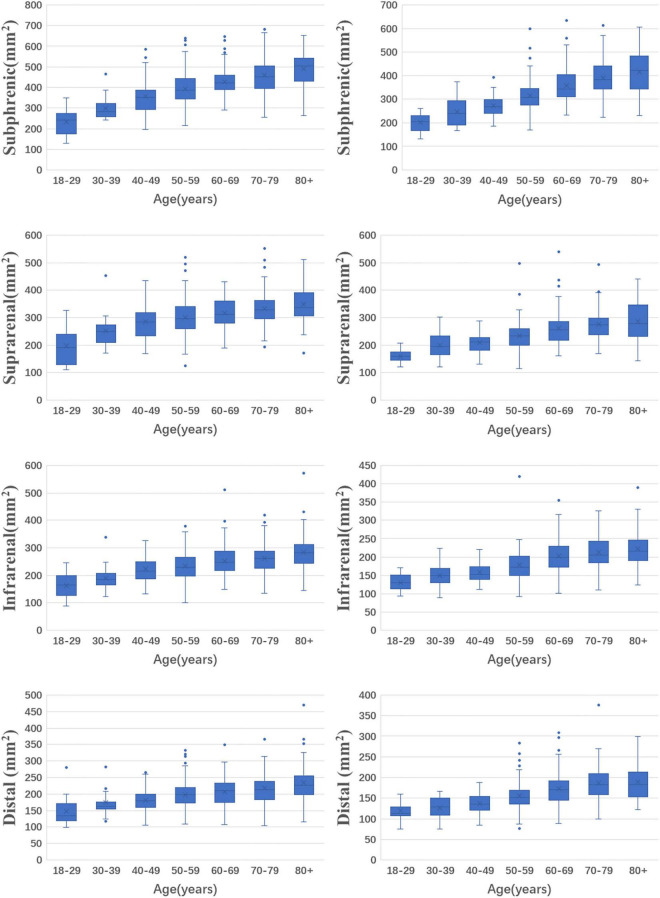
The distributions of abdominal aortic areas with age and sex in different levels. (The left column is for men and the right column is for women).

In males, the median areas of the subphrenic abdominal aorta, suprarenal abdominal aorta, infrarenal abdominal aorta, and distal abdominal aorta were 412.1, 308.0, 242.2 and 202.2 mm^2^, respectively. In females, the median areas of the subphrenic abdominal aorta, suprarenal abdominal aorta, infrarenal abdominal aorta, and distal abdominal aorta were 327.7, 243.4, 185.4 and 159.6 mm^2^, respectively. The areas of abdominal aorta at different levels were larger in males than in females and increased with age (*p* < 0.05 for all levels). The values for 5th, 25th, 50th, 75th, and 95th percentiles of the abdominal aortic areas at different levels are shown in Table 2. The distributions of abdominal aortic areas at different levels grouped by age and sex are shown in Figure 2.

**TABLE 2:** Age- and sex-specific percentiles of abdominal aortic areas (mm^2^) in the study sample.

Abdominal aorta	Age (years)	Male						Female					
		Percentiles						Percentiles					
		*n*	5th	25th	50th	75th	95th	*n*	5th	25th	50th	75th	95th
Subphrenic	18–29	21	132.2	173.2	241.2	273.8	344.5	20	133.6	166.3	205.1	231.3	260.8
	30–39	24	242.6	256.7	282.2	322.1	444.2	22	166.1	189.5	240.2	294.1	366.5
	40–49	51	223.6	293.7	350.8	386.6	531	55	190.5	240.1	267.4	299.1	350.4
	50–59	154	283.2	343.8	386.8	442.5	551.3	108	232.3	274.0	307.5	345.4	425.1
	60–69	162	324.9	388.3	421.8	457.7	547.8	115	262.3	311.1	343.9	403.6	457.2
	70–79	132	348.0	395.5	450.0	505.7	603.2	103	282.0	342.1	383.7	440.5	510.0
	80+	64	327.5	428.9	503.5	542.1	620.3	35	241.2	342.2	422.4	483.3	578.9
Suprarenal	18–29	21	111.5	128.5	191.8	239.9	325.9	20	121.0	145.7	160.9	179.7	205.8
	30–39	24	173.9	210.3	249.6	273.9	417.2	22	121.6	164.7	194.8	234.1	296.0
	40–49	51	176.7	233.8	283.3	317.8	421.5	55	148.7	181.4	210.6	227.9	282.2
	50–59	154	208.9	258.8	295.5	339.4	403.5	108	157.3	200.1	232.9	259.1	311.9
	60–69	162	226.7	280.7	313.0	359.4	405.7	115	187.4	218.2	255.1	285.2	373.0
	70–79	132	241.0	295.3	328.3	362.3	434.9	103	185.3	236.8	273.3	298.1	373.2
	80+	64	244.5	306.3	336.3	390.3	465.5	35	174.8	232.2	277.8	346.2	395.3
Infrarenal	18–29	21	91.9	126.4	165.2	199.7	243.3	20	92.9	113.8	129.9	150.3	170.8
	30–39	24	127.4	164.3	185.1	207.4	315.1	22	90.5	130.2	149.3	168.4	219.0
	40–49	51	150.2	187.3	215.1	249.7	306.5	55	118.7	138.2	152.3	173.9	210.9
	50–59	154	149.1	197.8	230.2	265.3	341.5	108	114.2	149.8	171.7	202.7	236.4
	60–69	162	171.6	217.2	247.1	288.1	344.6	115	135.4	171.1	199.8	229.6	278.3
	70–79	132	172.6	224.9	261.1	288.6	360.3	103	144.9	184.4	205.5	242.4	292.2
	80+	64	208.0	243.8	282.7	312.7	397.1	35	129.0	189.7	215.2	246.3	342.0
Distal	18–29	21	100.4	119.1	134.4	170.6	272.3	20	76.3	106.9	113.3	129.3	159.5
	30–39	24	119.7	154.4	163.3	176.4	285.1	22	76.9	109.1	128.3	150.3	165.7
	40–49	51	121.5	159.8	180.0	199.9	246.7	55	96.5	120.3	135.7	153.9	177
	50–59	154	136.9	172.3	197.2	219.3	273.8	108	109.1	136.2	151.9	169.4	217.2
	60–69	162	139.6	174.2	210.3	233.8	264.4	115	111.7	145.3	170.1	192.4	246.7
	70–79	132	151.8	183.2	213.4	238.3	302.9	103	116.9	158.6	182.5	209.1	253.4
	80+	64	157.3	198.1	226.2	255.5	362.6	35	122.5	152.8	183.2	213.4	293.5

Abdominal aortic areas were reduced from the subphrenic abdominal aorta to the distal abdominal aorta. The reduction was 50.9% in males and 51.3% in females.

### Expansion rate of abdominal aortic areas

The areas of abdominal aorta increased with age in both males and females (*p* < 0.001 for all aortic levels). Comparing the oldest group to the youngest group, the increase rate of the subphrenic abdominal aortic areas was the fastest (108.7% in males, 106.0% in females), followed by those of the suprarenal abdominal aortic areas were the second (75.3% in males, 72.7% in females), the infrarenal abdominal aortic areas were the third (71.1% in males, 65.7% in females), and distal abdominal aortic areas were the slowest (68.3% in males, 61.7% in females).

### Related influencing factors

Multiple linear stepwise regression analysis showed that the subphrenic abdominal aortic areas were significantly related to age (β = 0.544, *p* < 0.001), sex (β = 0.359, *p* < 0.001), and hypertension (β = 0.107, *p* < 0.001). The suprarenal abdominal aortic areas were related to age (β = 0.398, *p* < 0.001), sex (β = 0.383, *p* < 0.001), history of smoking (β = 0.074, *p* = 0.005), and hypertension (β = 0.111, p < 0.001). The infrarenal abdominal aortic areas were correlated with age (β = 0.420, *p* < 0.001), sex (β = 0.407, *p* < 0.001), and history of smoking (β = 0.055, *p* = 0.036). The distal abdominal aortic area was correlated with age (β = 0.463, *p* < 0.001), sex (β = 0.253, *p* < 0.001), and hypertension (β = 0.073, *p* = 0.013). The multiple linear regression Equation is as follows:


Area=subphrenic111.088+3.631X+170.728X+222.952X3(R=20.491)

Area=suprarenal121.582+1.981X+156.354X+217.752X+314.297X(R=20.385)4

Area=infrarenal84.400+1.743X+149.805X+28.791X(R=20.382)4

Area=distal87.150+1.221X+137.733X+211.404X(R=20.324)3

X_1_ is age (year), X_2_ is sex (male is 1 and female is 0), X_3_ is hypertension, and X_4_ is the history of smoking.

## Discussion

This study is the first to present the reference areas for the abdominal aorta in the normal Chinese adult population.

In our study, the respective median areas of the subphrenic abdominal aorta, suprarenal abdominal aorta, infrarenal abdominal aorta, and distal abdominal aorta were 412.1, 308.0, 242.2, and 202.2 mm^2^ in males. In females, the median areas of the subphrenic abdominal aorta, suprarenal abdominal aorta, infrarenal abdominal aorta, and distal abdominal aorta were 327.7, 243.4, 185.4, and 159.6 mm^2^, respectively. The area of the abdominal aorta decreased from proximal to distal. The different levels of abdominal aortic areas were lower in females than in males, which is consistent with published studies on abdominal aortic diameters (6–10).

The areas of the abdominal aorta were significantly increased with age. The most obvious is the subphrenic abdominal aorta. Comparing the subphrenic abdominal aortic areas between the 80+ years group and the 18–29 years group, the increase rate reached 108.7% in males and 106.0% in females. The distal abdominal aortic areas dilated the slowest, but also reached 68.3% in males and 61.7% in females. The abdominal aortic diameters also increased with age (7, 10–13). Our previous study showed a growth rate of 26.54% in the infrarenal abdominal aortic diameters compared between the 18–29 years old group and the 80–99 years old group (10). In this study, the infrarenal abdominal aortic area increased 71.1% in males and 65.7% in females. The increase rate of abdominal aortic areas was more obvious than the increase in abdominal aortic diameter. In a hemodynamic study of ruptured AAAs, an increase in the cross-sectional area of the flow lumen preceded an increase in AAA diameter (14). For the management of AAA, abdominal aortic areas may be a more sensitive indicator.

Our study found that abdominal aortic areas at different levels were positively correlated with age, sex, and hypertension. A history of smoking was also a positive factor of partial abdominal aortic area. This is similar to the factors associated with abdominal aortic diameter (15). Female sex was associated with 70.728, 56.354, 45.354, 37.733 mm^2^ reductions in the subphrenic abdominal aorta, suprarenal abdominal aorta, infrarenal abdominal aorta, and distal abdominal aorta, respectively. Hypertension was associated with 22.952, 17.752, and 11.404 mm^2^ increases in the subphrenic abdominal aorta, suprarenal abdominal aorta, and distal abdominal aorta, respectively. A history of smoking was associated with 14.297 and 8.791 mm^2^ increases in suprarenal abdominal aorta and infrarenal abdominal aorta, respectively. The subphrenic abdominal aorta, suprarenal abdominal aorta, infrarenal abdominal aorta, and distal abdominal aorta will expand 36.31, 19.81, 17.43, and 12.21 mm^2^, respectively, for 10 years.

The abdominal aortic diameter is currently a standard for the diagnosis and treatment of AAA, and it is an accessible and valid indicator. However, small diameter AAA can also rupture (16, 17). Therefore, finding other predictors of aneurysm expansion could identify high-risk patients, indicate early intervention, prevent disease progression, and reduce morbidity and mortality. In the past, limitations in medical technology precluded obtaining more image information, but the development and popularization of technology have provided increasingly abundant information from images. This information warrants exploration to improve the management of AAA.

At present, endovascular aneurysm repair (EVAR) is a mainstream method for the treatment of AAA. Compared with open surgery, EVAR is advantageous because it is less invasive and associated with fewer perioperative complications (18, 19). The size of the stent in the EVAR evaluation by the surgeon is mainly calculated based on the diameter of the abdominal aorta at the anchoring sites. After being released, the shape of a stent’s proximal end will be adjusted according to the shape of the anchoring site of the abdominal aorta. Poor-fitting between stents and the anchoring sites may lead to endoleak. In clinical practice, oversized stents are usually chosen to solve this problem (20). However, the choice of oversize remains controversial, and selecting the stent according to the area of the anchoring sites may be preferable. Further research is needed. This study was limited by the sample size of patients aged 18–39 years. This study is a single-center study, and it may not fully represent the Chinese population. A larger population-based study is needed.

## Conclusion

The abdominal aortic areas at different levels were larger in males than in females. Aging, hypertension, and smoking prompt the expansion of the abdominal aorta.

## Data availability statement

The raw data supporting the conclusions of this article will be made available by the authors, without undue reservation.

## Ethics statement

The studies involving human participants were reviewed and approved by the Taizhou Hospital of Zhejiang Province Ethic Committee. Written informed consent for participation was not required for this study in accordance with the national legislation and the institutional requirements.

## Author contributions

XW, SJ, JP, and WZ contributed to the conception and design. JP and JD performed the measurement of the abdominal aortic area. XW, SJ, QW, JL, FL, HC, DZ, and XZ performed the clinical data collection and interpretation. XW and SJ analyzed the datasets and wrote the manuscript. All authors read and approved the final manuscript.
